# *Plasmodium falciparum* field isolates drug susceptibility in Mali

**DOI:** 10.1093/jacamr/dlag015

**Published:** 2026-02-12

**Authors:** Fatoumata Ousmane Maiga, Laurent Dembele, Souleymane Dama, Ousmaila Diakite, Fatoumata Diallo, Fanta Sogore, Mohamed Maiga, François Dao, Niawanlou Dara, Mohamed Lamine Alhousseini, Oumar Bila Traore, Abdoulaye A Djimde

**Affiliations:** University of Sciences, Techniques, and Technologies of Bamako (USTTB), Faculty of Pharmacy, Parasites and Microbes Research and Training Center (P-MRTC), Point G. PBE: 1805, Bamako, Mali; Pathogens Genomic Diversity Network Africa (PDNA), Imm. Gwancoura, Sotuba, Bamako, Mali; University of Sciences, Techniques, and Technologies of Bamako (USTTB), Faculty of Pharmacy, Parasites and Microbes Research and Training Center (P-MRTC), Point G. PBE: 1805, Bamako, Mali; Pathogens Genomic Diversity Network Africa (PDNA), Imm. Gwancoura, Sotuba, Bamako, Mali; University of Sciences, Techniques, and Technologies of Bamako (USTTB), Faculty of Pharmacy, Parasites and Microbes Research and Training Center (P-MRTC), Point G. PBE: 1805, Bamako, Mali; University of Sciences, Techniques, and Technologies of Bamako (USTTB), Faculty of Pharmacy, Parasites and Microbes Research and Training Center (P-MRTC), Point G. PBE: 1805, Bamako, Mali; University of Sciences, Techniques, and Technologies of Bamako (USTTB), Faculty of Pharmacy, Parasites and Microbes Research and Training Center (P-MRTC), Point G. PBE: 1805, Bamako, Mali; University of Sciences, Techniques, and Technologies of Bamako (USTTB), Faculty of Pharmacy, Parasites and Microbes Research and Training Center (P-MRTC), Point G. PBE: 1805, Bamako, Mali; University of Sciences, Techniques, and Technologies of Bamako (USTTB), Faculty of Pharmacy, Parasites and Microbes Research and Training Center (P-MRTC), Point G. PBE: 1805, Bamako, Mali; University of Sciences, Techniques, and Technologies of Bamako (USTTB), Faculty of Pharmacy, Parasites and Microbes Research and Training Center (P-MRTC), Point G. PBE: 1805, Bamako, Mali; University of Sciences, Techniques, and Technologies of Bamako (USTTB), Faculty of Pharmacy, Parasites and Microbes Research and Training Center (P-MRTC), Point G. PBE: 1805, Bamako, Mali; University of Sciences, Techniques, and Technologies of Bamako (USTTB), Faculty of Pharmacy, Parasites and Microbes Research and Training Center (P-MRTC), Point G. PBE: 1805, Bamako, Mali; University of Sciences, Techniques, and Technologies of Bamako (USTTB), Faculty of Pharmacy, Parasites and Microbes Research and Training Center (P-MRTC), Point G. PBE: 1805, Bamako, Mali; University of Sciences, Techniques, and Technologies of Bamako (USTTB), Faculty of Pharmacy, Parasites and Microbes Research and Training Center (P-MRTC), Point G. PBE: 1805, Bamako, Mali; Pathogens Genomic Diversity Network Africa (PDNA), Imm. Gwancoura, Sotuba, Bamako, Mali

## Abstract

**Background and objectives:**

The emergence and spread of antimalarial drug resistance threaten malaria control efforts in sub-Saharan Africa. Monitoring the susceptibility of circulating *Plasmodium falciparum* isolates is essential to inform national treatment guidelines and guide the development of new therapies. To assess the *ex vivo* susceptibility of *P. falciparum* field isolates to 14 antimalarials, including withdrawn/unused and currently used drugs, and next-generation agents in Mali.

**Methods:**

Twenty-six isolates collected from patients with uncomplicated malaria at three endemic sites (Faladje, Kolle and Bougoula-Hameau) were cultured *ex vivo* under standardized conditions. Parasites were exposed to 14 drugs, including tafenoquine, N-desethyl-amodiaquine, chloroquine, dihydroartemisinin, lumefantrine, pyronaridine, quinine, sulfadoxine, pyrimethamine, amodiaquine, atovaquone, GNF179, KDU691 and cabamiquine. Susceptibility was measured using fluorescence-based assays with SYBR Green I and Mitotracker dyes, and IC_50_ values were derived from dose–response curves.

**Results:**

Tafenoquine showed a very low potency (IC_50_ > 1500 nM). Chloroquine exhibited marked inter- and intra-site variability (IC_50_ ∼50–1300 nM), while N-desethyl-amodiaquine potently inhibited isolates (median IC_50_ < 20 nM in Faladje and Bougoula-Hameau). Current frontline drugs, dihydroartemisinin (median IC_50_ < 6 nM), lumefantrine (median IC_50_ < 50 nM) and pyronaridine (median IC_50_ < 10 nM), remained highly potent. Quinine showed variable efficacy (IC_50_ ∼75–1000 nM). Chemoprevention agents sulfadoxine and pyrimethamine displayed high IC_50_ values (median IC_50_ > 1000 and >2000 nM). Atovaquone and amodiaquine consistently inhibited all isolates (IC_50_ < 10 nM). Next-generation compounds cabamiquine and GNF179 demonstrated consistently strong activity (IC_50_ < 10 nM), while KDU691 showed moderate activity (median IC_50_ 18–22 nM).

**Conclusions:**

While current frontline therapies remain effective, reduced activity of chemopreventive antimalarials supports the need for continued surveillance to detect early signs of resistance in Mali. The potent activity of next-generation candidates (cabamiquine and GNF179) supports their potential for further clinical development and field deployment.

## Introduction

Malaria, predominantly caused by *Plasmodium falciparum*, continues to be a significant public health challenge, especially in sub-Saharan Africa. Despite extensive control efforts, the burden remains very high for the WHO African Region, which alone accounted for approximately 265 million of these cases (94%) and 579 000 deaths (95%).^1^ Mali accounted for approximately 3% of global malaria cases, roughly 8.475 million cases and 3% of global deaths (17 370 fatalities) in 2024.^1^ Chemoprevention remains central to malaria prevention, while effective case management is key to effectively controlling uncomplicated and severe malaria through the use of artemisinin-based combination therapies (ACTs) in oral forms and parenteral administration of artesunate,^2^ respectively. However, the emergence and spread of drug resistance threatens the efficacy of existing regimens and poses a significant barrier to elimination.^3^

The evolution of antimalarial drug resistance has historically undermined control programs. Chloroquine, which was the main treatment for malaria from the 1950s until the late 1990s,^4^ became ineffective because of widespread resistance,^5^ leading to its global withdrawal in the 2000s. Later, in response to sustained sulfadoxine–pyrimethamine’s use as first-line treatment, *P. falciparum* parasites developed resistance by selecting for mutations in the dhfr and dhps genes.^6–8^ Currently, ACTs are the frontline treatment for uncomplicated malaria,^9^ combining fast-acting artemisinin derivatives with long-acting partner drugs to enhance efficacy and reduce the risk of resistance development.^10^ However, the emergence of artemisinin partial resistance^11^ poses a new threat to malaria control efforts.

Initially identified in Southeast Asia,^12–14^ artemisinin partial resistance has since been detected in several countries of sub-Saharan Africa, including Rwanda and Uganda.^15,16^ Although artemisinin partial resistance has not yet been reported in Mali, the presence of resistant parasites in neighbouring regions and substantial cross-border human movement increase the risk of artemisinin partial resistance in Mali.^17^ Promptly detecting imported or newly emerged resistance cases and trends requires surveillance and monitoring of therapeutic agent efficacy, local field isolates’ susceptibility and drug resistance molecular markers.

Field isolates of *P. falciparum* are ideal for tracking and understanding the dynamics of parasite resistance. Unlike laboratory strains, which often do not reflect the genetic and phenotypic diversity of natural populations, field isolates give a real picture of evolutionary events in parasite populations, including emerging resistance mechanisms. *In vitro* drug susceptibility assays provide an estimation of IC_50_, defined as the concentration of a compound required to inhibit 50% of parasite growth, and provide phenotypic data on drug efficacy. While molecular surveillance is generally the first-line approach for resistance monitoring, IC_50_ assays remain essential for confirming the impact of mutations and identifying reduced susceptibility^18^ when molecular markers are absent or not yet validated.^19^ Although not always directly equivalent to *in vivo* therapeutic efficacy, standardized IC_50_ thresholds have been established for several antimalarial drugs based on correlations with treatment failures or validated molecular resistance markers. Importantly, IC_50_ cutoffs differ by drug class,^20^ pharmacodynamics and mechanism of action.^19^ For instance, atovaquone is typically used with a susceptibility threshold around 30 nM^21^ (Table 1), chloroquine with IC_50_ values above 100 nM,^22,23^ while sulfadoxine and pyrimethamine exhibit IC_50_ values frequently in the micromolar range, even in susceptible isolates, due to their enzymatic targets.^24,25^ In contrast, artemisinin derivatives usually show IC_50_ values <10 nM^26^ and lack a universally accepted cutoff because clinical resistance currently manifests through delayed clearance^17,26,27^ (Table 1).

**Table 1.** IC_50_ values and thresholds for indicative ranges for antimalarial drug susceptibility

Drug	Median IC_50_ range (nM) (all sites)	Reported cutoff for reduced susceptibility or resistance	# of isolates with high IC_50_	Overall interpretation for our data
Tafenoquine	1493–2428	No standardized cutoff	na	High IC_50_s
Chloroquine	11–1316	>100 nM reduced susceptibility^22,23^	13	Mixed; resistance still common
N-desethyl-amodiaquine	2–1069	>60 nM reduced susceptibility^46^	5	Generally potent; site-specific reduced susceptibility
Dihydroartemisinin	0.5–5	No validated cutoff^46^	na	Highly potent
Lumefantrine	10–148	>150 nM reduced susceptibility^75^	0	Potent
Pyronaridine	2–17	> 15 nM reduced susceptibility^76^	1	Highly potent
Quinine	11–2384	>800–1000 nM reduced susceptibility^77^	8	Variable; decreased susceptibility in one site
Sulfadoxine	435–5623	No validated cutoff		Reduced susceptibility
Pyrimethamine	17–5919	>2000 nM resistance^25^	16	Reduced susceptibility
Amodiaquine	1–10	>80 nM reduced susceptibility^34^	0	Highly potent
Atovaquone	1–680	>28 nM resistance^21,34^	2	Highly potent overall
GNF179	3–10	No validated cutoff	na	Highly potent
KDU691	11–37	No validated cutoff	na	Highly Potent
Cabamiquine	1–4	No validated cutoff	na	Highly potent

Table 1 presents the IC_50_ value ranges in our study, which were interpreted using the ‘Reported Cutoff’ indicated in the literature for reduced susceptibility or resistance. The number of isolates with high IC_50_ denotes the number of isolates in this study exceeding the drug-specific cutoff. For drugs without a standardized cutoff, this field is listed as ‘na’ (not applicable).

This study evaluates the *ex vivo* efficacy of 14 antimalarial drugs (classified as withdrawn, unused, currently used, or lead advanced candidate antimalarial drugs) against *P. falciparum* field isolates collected in three Malian endemic sites: Kolle, Bougoula-Hameau and Faladje. The three study sites differ in malaria transmission intensity according to national surveillance data and previous longitudinal studies. Faladje is a seasonal transmission area (July–October).^28^ Bougoula-Hameau also experiences intense seasonal transmission (July–November).^29,30^ In contrast, Kolle represents a meso-endemic setting.^31^ Thus, these sites, with their distinct ecological and transmission dynamics, offer a robust framework for assessing geographic variations in drug susceptibility in the country. By analysing IC_50_ values across these regions, this research provides critical insights into the current efficacy of antimalarial drugs in Mali, aiming to inform treatment strategies, especially to strengthen surveillance programs by providing susceptibility profiles of three ecologically different sites.

## Methods

### Sample collection and preparation

**Figure 1.**
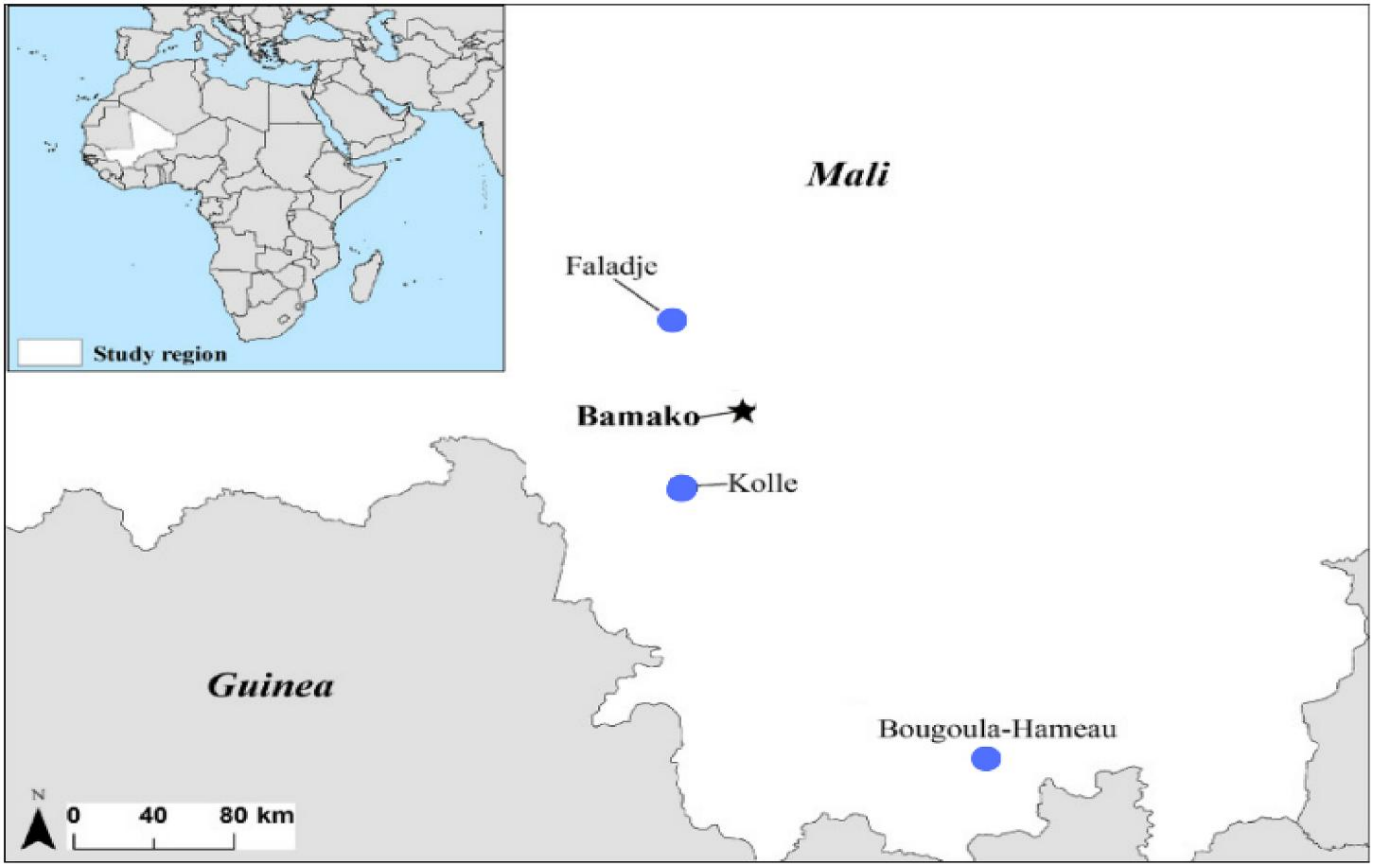
Map of sample collection sites. Study sites are shown with blue circles in the map, and Bamako (the capital city of Mali) with a star, where the *ex vivo* assays were performed. The three study sites were all located in the southern part of Mali.

In the current study, a total of 26 field isolates of *Plasmodium falciparum* were collected from patients diagnosed with uncomplicated malaria from three malaria-endemic sites (Figure 1) in Mali: Faladje (*n* = 10), Kolle (*n* = 10) and Bougoula-Hameau (*n* = 6). For the three study sites, the isolates were collected in the field between September and October 2023.

For blood sample collection, patients were enrolled using inclusion criteria that required parasitemia levels above 20 000 trophozoites/µL and no prior antimalarial drug use in the past 14 days.

Approximately 6 mL of venous blood was collected into acid-citrate-dextrose tubes. Blood samples were transported to the laboratory under cold chain conditions (4°C–8°C) within 6 h to preserve parasite viability. Upon arrival, the blood underwent further processing. A thin smear was prepared and stained using Giemsa 15% to confirm parasitemia levels. Blood samples were washed three times with incomplete RPMI 1640 medium (RPMI 1640 medium + 25 mM HEPES + 2 g/L glucose + 0.2% sodium bicarbonate + 50 µg/mL gentamicin, without Albumax and hypoxanthine) to remove leukocytes. Washed erythrocytes were cultured in complete RPMI made of incomplete RPMI 1640 medium supplemented with 50 µg/mL hypoxanthine and 0.5% Albumax. Parasites were synchronized to the ring stage using a 5% sorbitol solution to ensure uniform development. Washed blood was adjusted to 2% haematocrit for drug susceptibility testing.

### Drug selection

These isolates were *ex vivo* cultured in the laboratory and exposed to a panel of 14 antimalarial drugs representing three categories: (i) drugs never or no longer used in Mali (chloroquine, tafenoquine and N-desethyl-amodiaquine), (ii) drugs currently in use (dihydroartemisinin, lumefantrine, pyronaridine, quinine, sulfadoxine, pyrimethamine, amodiaquine and atovaquone) and (iii) lead advanced candidate antimalarial drugs (GNF179, cabamiquine and KDU691). The currently used drugs were further divided into two sub-groups: drugs used in the cure (dihydroartemisinin, lumefantrine, pyronaridine and quinine) and drugs used in malaria prevention (sulfadoxine, pyrimethamine, amodiaquine and atovaquone). Stock solutions of each drug were prepared in 100% dimethyl sulfoxide (DMSO) and stored at −20°C. Working dilutions of 10 000 nM for each compound were freshly prepared in RPMI 1640 medium to achieve final 1/3 serial diluted concentrations in 08 duplicated wells with a final DMSO concentration of 0.002%.

### Efficacy testing

**Figure 2.**
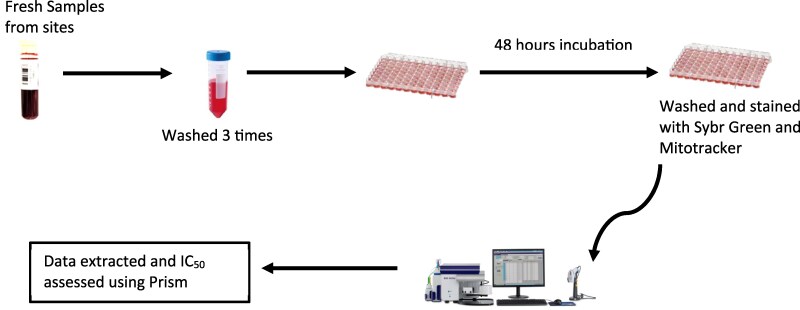
Workflow of the drug assay and data processing/analysis. Freshly collected field isolates were washed, synchronized and exposed to serial dilutions of each antimalarial compound in 96-well plates. Cultures were incubated for 48 h at 37°C in the presence of the test antimalarial drug. After incubation, cells were washed and stained with SYBR Green I and Mitotracker dyes, and IC_50_ values were calculated from dose–response curves.

Blood samples were processed at the Parasites and Microbes Research and Training Center (PMRTC), University of Sciences, Techniques and Technologies of Bamako (USTTB), Bamako, Mali. The *ex vivo* susceptibility of field isolates to the selected drugs was assessed using fluorescence-based assays, as reported previously.^32^ SYBR Green I and Mitotracker dyes were used to quantify parasite growth inhibition. Serial dilutions (1 in 3) of each drug were dispensed into 96-well plates, and each concentration was tested in duplicate wells. Cultures with 1% parasitemia and 2% haematocrit were added to each well. Plates were then incubated for 48 h (Figure 2) at 37°C in a controlled environment (5% CO_2_, 5% O_2_, 90% N_2_). To adjust the blood sample to a final haematocrit of 2%, we first measured the volume of packed red blood cells after the last wash and resuspended this volume in complete RPMI 1640 medium accordingly. Thus, to achieve a haematocrit of 2% for example, in 10 mL final media, this will require 0.2 mL of packed red blood cells plus 9.8 mL of media. After the incubation, plates were analysed using a flow cytometer (Brand BD), where SYBR Green I and Mitotracker staining enabled the detection and quantification of parasite DNA and mitochondrial activity, respectively.

### Data analysis

IC_50_ values were determined by fitting dose–response curves and compared across sites using Kruskal–Wallis tests in GraphPad Prism software (Figure 2). The one-way ANOVA and Tukey’s *post hoc* tests were performed to identify any significant site-specific differences in drug efficacy. Results with a *P* value < 0.05 were considered statistically significant.

### Ethical considerations

The current study protocol was reviewed and approved by our institutional ethical committee of the Faculties of Medicine-Odonto-Stomatology and Pharmacy, University of Science, Techniques and Technologies of Bamako, Mali, with the reference N° 2023/03/CE/USTTB. Only participants or their parent/guardian who provided written informed consent, plus children able to understand the study and who gave assent, were enrolled in this study. All patients with malaria who consented to participate in the study were enrolled and treated using recommended artemether–lumefantrine (AL) to clear the parasites as recommended by the ethics committee.

## Results

### Susceptibility of *P. falciparum* field isolates collected from three different sites in Mali to never-used or abandoned drugs tafenoquine, chloroquine and N-desethyl-amodiaquine

**Figure 3.**
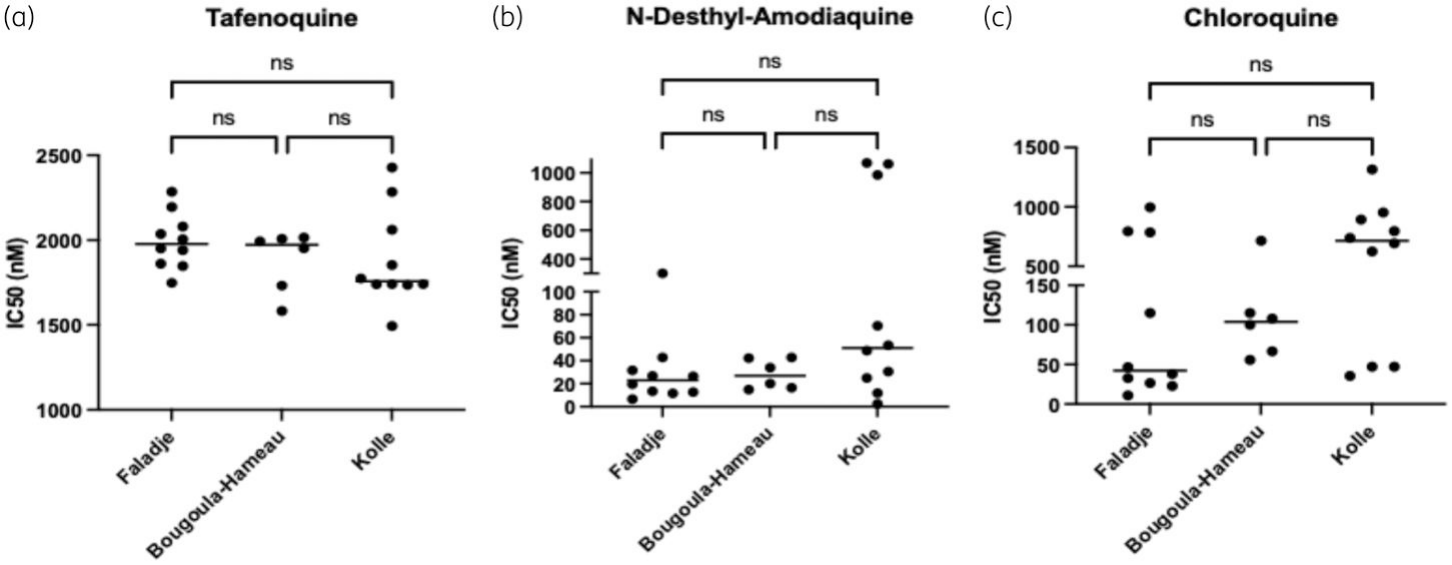
*In vitro* susceptibility of *P. falciparum* field isolates to tafenoquine, N-desethyl-amodiaquine and chloroquine across three study sites in Mali. (a) IC_50_ of tafenoquine; (b) IC_50_ of N-desethyl-amodiaquine; (c) IC_50_ of chloroquine.

Tafenoquine displayed IC_50_ values >1500 nM (Figure 3a) across all three study sites. In contrast, N-desethyl-amodiaquine showed lower IC_50_ values in Faladje and Bougoula-Hameau (Median IC_50_ < 40 nM) compared to Kolle, where several not susceptible parasites showed micromolar IC_50_ values (∼1000 nM; Figure 3b). Chloroquine, which was withdrawn in Mali in 2005–6,^33^ demonstrated a broader range of IC_50_ values (∼25 to ∼1000 nM), with considerable inter- and intra-site variability, indicating that many field isolates remained non-susceptible to chloroquine. The isolates showing the high chloroquine IC_50_ values (500–1300 nM) were observed in all three sites (Figure 3c). A subset of individual parasite isolates in Faladje (6/10) and Kolle (3/10) showed relatively low IC_50_ values (< 50 nM) in the range of chloroquine-susceptible parasites (Figure 3c).

### Susceptibility of *P. falciparum* field isolates to currently used antimalarial drugs in Faladje, Bougoula-Hameau and Kolle, Mali’s field sites

**Figure 4.**
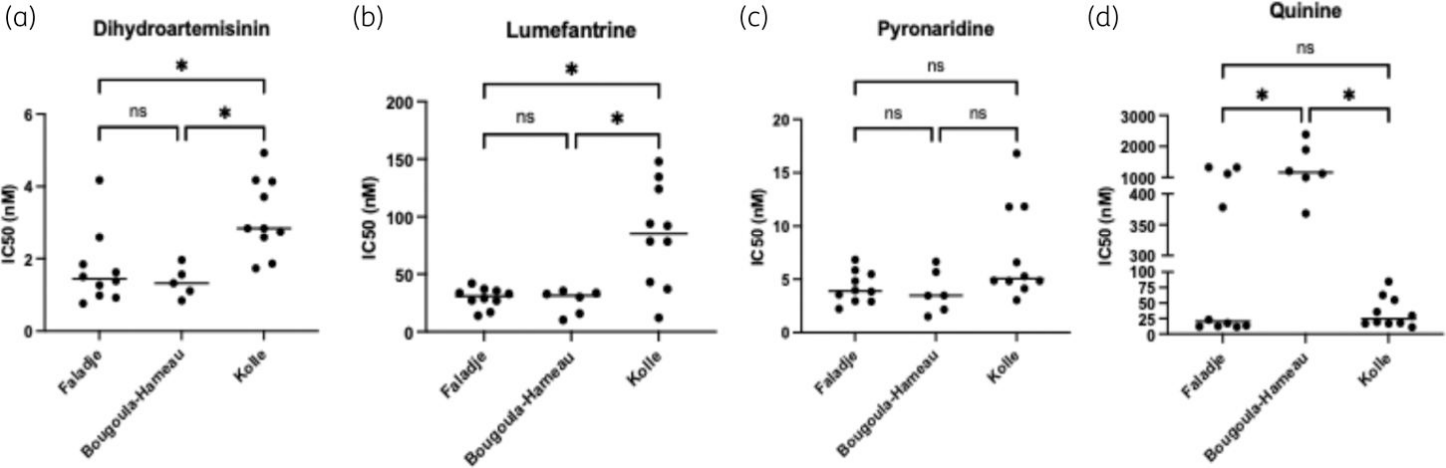
*In vitro* susceptibility of *P. falciparum* field isolates to dihydroartemisinin, lumefantrine, pyronaridine and quinine across three study sites in Mali. (a) IC_50_ of dihydroartemisinin; (b) IC_50_ of lumefantrine; (c) IC_50_ of pyronaridine; (d) IC_50_ of quinine.

All field isolates showed IC_50_ values < 6 nM for dihydroartemisinin, as we expected (Figure 4a). The compared IC_50_ median across field sites displayed significantly higher (*P* value < 0.05) IC_50_ in Kolle compared to Faladje and Bougoula-Hameau (Figure 4a). Similarly, lumefantrine showed low IC_50_ values (< 50 nM) in Faladje and Bougoula-Hameau, while Kolle had a significantly higher IC_50_ median of nearly 150 nM (Figure 4b). For pyronaridine, the median IC_50_ values of the susceptibility in the three distinct site isolates were similar (<10 nM). Thus, field isolates’ susceptibility did not differ significantly across the three study sites (*P* value > 0.05) (Figure 4c). Unlike the other drugs currently used in Mali, quinine displayed a wide range of IC_50_ values across the sites, with significantly higher IC_50_ median values in Bougoula-Hameau (> 1000 nM) compared to Faladje and Kolle (both < 75 nM; *P* value < 0.05). Some isolates displayed significantly decreased susceptibility to quinine of field isolates from Faladje and Bougoula-Hameau (Figure 4d). Interestingly, *P. falciparum* field isolates from Kolle were all fully susceptible to quinine (Figure 4d).

### Malaria preventive chemotherapy compounds activities against the field isolates *P. falciparum* parasite from Faladje, Bougoula-Hameau and Kolle

**Figure 5.**
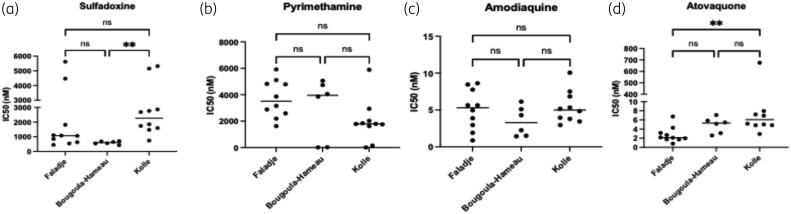
*In vitro* susceptibility of *P. falciparum* field isolates to sulfadoxine, pyrimethamine, amodiaquine and atovaquone across three study sites in Mali. (a) IC_50_ of sulfadoxine; (b) IC_50_ of pyrimethamine; (c) IC_50_ of amodiaquine; (d) IC_50_ of atovaquone.

Sulfadoxine displayed IC_50_ median values > 1000 and > 2000 nM, respectively, in Faladje and Kolle (Figure 5a). The higher IC_50_ values in Kolle resulted in a significant difference in the IC_50_ median compared to Bougoula-Hameau (*P* value < 0.01) (Figure 5a). Its partner drug, pyrimethamine, displayed intra-site variability across all three sites, with IC_50_ values ranging from 100 to 6000 nM (Figure 5b). Pyrimethamine IC_50_ median values in Faladje (2800 nM) and Bougoula-Hameau (4000 nM) nearly doubled that of Kolle (1800 nM). Several isolates exhibited cumulative reduced susceptibility, with elevated IC_50_ values to multiple antimalarial drugs, including pyrimethamine and chloroquine, regardless of the study site (Table 2). In contrast, amodiaquine showed consistent activity profiles against *P. falciparum* field isolates across the three sites, with low IC_50_ values (<10 nM) (Figure 5c). Similarly, atovaquone IC_50_ values were below 10 nM, except for one individual *P. falciparum* field isolate in Kolle. However, the median IC_50_ in Kolle (6 nM) of atovaquone against *P. falciparum* field isolates was significantly higher (*P* value < 0.01) and three times greater than that of Faladje (2 nM) (Figure 5d).

**Table 2.** Isolate-level antimalarial susceptibility profiles using established IC_50_ thresholds

Study site	Isolate ID	Number of drugs with elevated IC_50_	High IC_50_ drug values (nM)
Bougoula-Hameau	Isolate B1	3	PYR: 3854 nM; CQ: 115.1 nM; QN: 1893 nM
Bougoula-Hameau	Isolate B2	2	PYR: 4055 nM; CQ: 100 nM
Bougoula-Hameau	Isolate B3	2	CQ: 107.9 nM; QN: 1122 nM
Bougoula-Hameau	Isolate B4	2	CQ: 715 nM; QN: 2384 nM
Bougoula-Hameau	Isolate B5	2	PYR: 5057 nM; QN: 1000.3 nM
Bougoula-Hameau	Isolate B6	2	PYR: 4739 nM; QN: 1200 nM
Faladje	Isolate F1	3	PYR: 4820 nM; CQ: 115.1 nM; QN: 1329.4 nM
Faladje	Isolate F2	1	PYR: 3155 nM
Faladje	Isolate F3	2	PYR: 2587 nM; QN: 1322 nM
Faladje	Isolate F4	2	PYR: 4788 nM; CQ: 785 nM
Faladje	Isolate F5	1	PYR: 5919 nM
Faladje	Isolate F6	2	PYR: 5111 nM; QN: 1118 nM
Faladje	Isolate F7	1	PYR: 2884 nM
Faladje	Isolate F8	3	PYR: 2193 nM; DES-A: 301.1 nM; CQ: 996.4 nM
Faladje	Isolate F9	0	
Faladje	Isolate F10	2	PYR: 3864 nM; CQ: 793.7 nM
Kolle	Isolate K1	0	
Kolle	Isolate K2	0	
Kolle	Isolate K3	2	DES-A: 1062 nM; CQ: 693.8 nM
Kolle	Isolate K4	2	DES-A: 984.7 nM; CQ: 1316 nM
Kolle	Isolate K5	3	PYR: 5887 nM; DES-A: 1069.1 nM; CQ: 952.7 nM
Kolle	Isolate K6	3	PYR: 2026 nM; DES-A: 70.43 nM; CQ: 796.9 nM
Kolle	Isolate K7	0	
Kolle	Isolate K8	2	PN: 16.8 nM; CQ: 624.9 nM
Kolle	Isolate K9	2	ATQ: 42.35 nM; CQ: 893.1 nM
Kolle	Isolate K10	3	PYR: 2942 nM; ATQ: 676 nM; CQ: 738.51 nM

Table 2 presents the number of drugs with elevated IC_50_ values for each isolate, along with the corresponding IC_50_ values for those drugs. Elevated IC_50_s were identified by comparing observed values to literature-derived thresholds for reduced susceptibility. The following thresholds were applied: quinine (QN) > 800 nM, chloroquine (CQ) > 100 nM, N-desethyl-amodiaquine (DES-A) > 60 nM, lumefantrine (LUM) > 150 nM, pyronaridine (PN) > 15 nM, pyrimethamine (PYR) > 2000 nM, amodiaquine (AMQ) > 80 nM and atovaquone (ATQ) > 28 nM.

### Lead advanced discovery antimalarial agents candidates potently inhibited *P. falciparum* field isolates from Bougoula-Hameau, Faladje and Kolle

**Figure 6.**
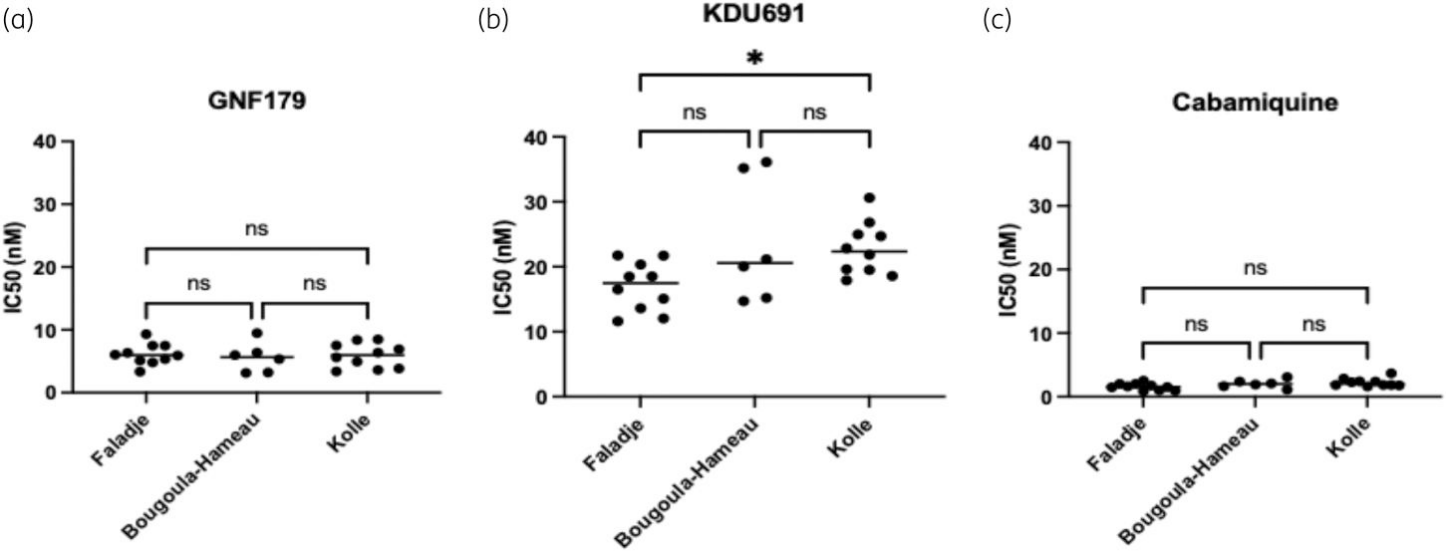
*In vitro* susceptibility of *P. falciparum* field isolates to lead advanced discovery of antimalarial agents across three study sites in Mali. (a) IC_50_ of GNF179; (b) IC_50_ of KDU691; (c) IC_50_ of cabamiquine.

The imidazolopiperazine GNF179, a close analogue of KAF156 in Phase III clinical trials, demonstrated high and consistent potency against *P. falciparum* field isolates across all three study sites, with an IC_50_ median of 5–10 nM (Figure 6a). Similarly, the *Plasmodium PI(4)K* inhibitor (KDU691) potently inhibited *P. falciparum* field isolates with IC_50_ values < 40 nM in all sites (Figure 6b). KDU691 displayed an IC_50_ median in Kolle of 22 nM, which was statistically higher (*P* value < 0.05) when compared to that of Faladje (18 nM) (Figure 6b). Cabamiquine has shown the most highly inhibitory activity regardless of the parasite collection sites. Thus, cabamiquine displayed a nanomolar range IC_50_ < 5 nM across the three sites (Figure 6c). Finally, parasites from the three sites appeared to be fully susceptible to all the lead-advanced discovery antimalarial agent candidates (Figure 6).

## Discussion

This study was conducted to assess the current drug susceptibility profile of *Plasmodium falciparum* to antimalarial drugs used to prevent and cure malaria, along with those no longer in use in Mali and those candidates under clinical investigation. To assess the direct resistance profile of field-isolated parasites, we used the drug-related IC_50_, which is a quantitative measure of drug efficacy and parasite susceptibility. In this study, we used field isolates of *Plasmodium falciparum* from the three different endemic sites, which can be representatives of the circulating parasite populations in Mali. This approach aimed to facilitate the detection of early-stage resistance, which can inform the usefulness of the current study-targeted drugs.

Tafenoquine has not been approved for use in Mali. Despite its absence in the field, tafenoquine displayed weak potency against *P. falciparum* field isolates with high median IC_50_ values >1500 nM across all study sites, as also reported earlier in other studies using field isolates.^34^ Low values of IC_50_ (below 100 nM) were reported in the early 2000s when tafenoquine was introduced in some sub-Saharan countries like Ghana,^35^ Gabon, Djibouti and Senegal.^34^ Tafenoquine is also known for its efficacy against multidrug-resistant strains.^36^ High values of tafenoquine IC_50_ can be observed in multidrug-resistant *P. falciparum* strains and Indochina strains resistant to chloroquine.^37^ In Mali field isolates, tafenoquine IC_50_ values were uniformly high across all sites, while chloroquine IC_50_ values were highly variable (50–1300 nM). Thus, regardless of the IC_50_ value of chloroquine, we observed high tafenoquine IC_50_ across all sites. This observation is consistent with previous studies showing that tafenoquine exhibits relatively high IC_50_ and IC_90_ values in standard *in vitro* blood-stage assays, including against multidrug-resistant *P. falciparum* isolates from Southeast Asia and African field isolates.^34,37,38^ These IC values often exceed proposed *in vivo* minimum inhibitory concentrations, suggesting that tafenoquine’s clinical efficacy is not well predicted by short-term blood-stage *in vitro* assays.^37^ This could also be explained by the fact that tafenoquine is a prodrug like primaquine, an 8-aminoquinoline antimalarial that requires metabolic activation, with its efficacy coming from its active metabolites.^39^ The lower IC_50_ values for N-desethyl-amodiaquine (never been used as an independent formulated therapeutic agent in Mali) in Faladje and Bougoula-Hameau are consistent with susceptibility in the previous report.^40^ The decreased susceptibility in Kolle reaching IC_50_ 1000 nM could indicate emerging resistance, calling for further investigations on these strains and a larger sample size investigation.

Chloroquine showed considerable inter- and intra-site variability, with high IC_50_ values (500–1300 nM) in all sites but lower values (<50 nM) in a subset of individual parasite isolates in Faladje and Kolle. This variability suggests that while chloroquine resistance is still widespread, there may be new emerging spots of susceptibility, possibly due to the withdrawal of chloroquine since 2006.^33^ The few high IC_50_ values observed across the three study sites support the importance of continuous monitoring of chloroquine resistance to assess susceptibility, which can be restored due to its prolonged withdrawal.^41–43^

Artemisinin is the current frontline therapeutic tool against malaria. When its active component, dihydroartemisinin, was tested against *P. falciparum* field isolates from all sites, the parasites were fully susceptible with IC_50_ < 5 nM. This full susceptibility of the parasite is in good agreement with Artemisinin’s continued efficacy in Mali, as its derivatives act as the fast-acting core components of ACTs, while partner drugs such as lumefantrine provide the long-acting activity needed to clear residual parasites and prevent recrudescence^19^ of first-line treatments in Mali.^29^ However, the higher median IC_50_ in Kolle compared to Faladje and Bougoula-Hameau suggests differences in field isolate susceptibility. Globally, the low values of IC_50_ median are consistent with the current level of artemisinin and derivatives’ susceptibility in other West African countries.^44–47^ Lumefantrine also showed relatively low IC_50_ values (< 50 nM) in Faladje and Bougoula-Hameau, with a higher IC_50_ median in Kolle (80 nM), which may be signs of concerns about reduced susceptibility of the *Plasmodium falciparum* to lumefantrine as reported previously.^48^ Higher lumefantrine IC_50_ values in Kolle may result from local selection pressure driven by extensive AL use and the associated prevalence of *Pfmdr1* alleles linked to reduced lumefantrine susceptibility in West African parasite populations.^49,50^ Pyronaridine, with consistently low IC_50_ values (∼5 nM) across all sites, appears to be effective, supporting its use as part of combination therapies. Quinine displayed a wide range of IC_50_ values, with significantly higher IC_50_ median values in Bougoula-Hameau (>1000 nM) compared to Faladje and Kolle (<25 nM). Such a wide range of variability of IC_50_ values may be due to the long-term historical use of quinine in the field, causing diversity in the parasite response. Although elevated quinine IC_50_ values have been reported, clinically confirmed quinine resistance has not been documented in Mali. The high IC_50_ values in Bougoula-Hameau, a place that dominates the malaria burden in Mali, could be explained by the heavy use of quinine in complicated malaria case management, as quinine was the first treatment option.^51^

Current malaria prevention tools in Mali, including Seasonal Malaria Chemoprevention (SMC) in children under five, Intermittent Preventive Treatment in pregnancy with SP (IPTp-SP) for pregnant women, Intermittent Preventive Treatment in infants (IPTi) mainly use sulfadoxine and pyrimethamine. The high IC_50_ median values for sulfadoxine in Faladje and Kolle (>1000 and >2000 nM, respectively) are a good indication of sulfadoxine resistance, consistent with previous reports showing an increase in sulfadoxine-pyrimethamine (SP) resistance in Mali.^24,52^ Pyrimethamine also showed substantial intra-site variability, with median IC_50_ values in Faladje and Bougoula-Hameau nearly double that of Kolle. Combined, the high values of IC_50_ for both pyrimethamine and sulfadoxine raise concerns about using both in intermittent preventive treatment in pregnant women^53^ and the seasonal malaria chemoprevention in children under five^54^ in Mali. The consistently elevated IC_50_ values that we observed for sulfadoxine and pyrimethamine are biologically plausible in a setting where antenatal folate is widely used, because exogenous folate can reduce the antimalarial activity of antifolates.^55–57^  *In vitro*, physiological concentrations of folic acid markedly reduce Sulfadoxine potency and, to a lesser extent, pyrimethamine, by resupplying the parasite’s folate pool and bypassing DHPS/DHFR blockade.^58,59^  *In vivo*, trials and observational studies in pregnant women show that high-dose folic acid (5 mg/day) and higher circulating folate levels are associated with increased SP treatment failure.^55–57^ Hence, the WHO and partners’ recommendation for using iron plus low-dose folic acid (0.4 mg/day) with IPTp-SP.^60–62^

Unlike sulfadoxine and pyrimethamine, amodiaquine and atovaquone showed very low IC_50_ values across all sites, compared to their susceptibility reference cutoff value of 30 nM.^63^ The Low IC_50_ values for both amodiaquine and atovaquone (Figure 5c and d) suggest that these drugs remain effective for *P. falciparum* malaria prevention, as observed in other studies conducted years ago on *P. falciparum* strains from African countries.^64^ Amodiaquine, demonstrating consistent efficacy with IC_50_ values below 10 nM, may further support its use in combination therapies. However, the higher median IC_50_ for atovaquone in Kolle (6 nM) compared to Faladje (2 nM) (Figure 5d) calls for continued monitoring for early detection of emerging resistance, even though the drug remained fully active in this study. The relatively low IC_50_ values of N-desethyl-amodiaquine and amodiaquine support the continued use of these drugs as part of combination therapies in Mali, such as with lumefantrine in the AL first-line combination. However, the extensive use of lumefantrine could potentially accelerate the emergence of parasite strains resistant not only to lumefantrine but also to amodiaquine due to cross-selection pressure with mutations on *Pfmdr1* and *Pfcrt* genes.^65–67^

Having tested and evaluated the current therapeutic susceptibility profile, we set out to establish the next-generation antimalarial drug candidate activity against *P. falciparum* field isolates’ susceptibility across the three field sites (Figure 6). Imidazolopiperazine GNF179, a close analogue of KAF156 and cabamiquine, the *Plasmodium* elongation factor 2 inhibitor, respectively in Phase III and Phase II clinical development, potently inhibited all isolates from all sites (median IC_50_ ∼ 5 nM) (Figure 6), suggesting that these drugs under investigation are promising candidates against *P. falciparum* malaria. Early development studies on these drugs confirmed their *ex vivo* and *in vitro* efficacy against *P. falciparum* malaria parasites.^32,68,69^ Furthermore, KAF156, the parent analogue of GNF179, has proven its *in vivo* efficacy against *P. falciparum* malaria and is currently in Phase IIb trials.^70–73^ Even less potent than GNF179 and cabamiquine, the *PI4K inhibitor* KDU691 displayed also potent inhibitory activity (IC_50_ median 50 nM) (Figure 6b) against *P. falciparum* malaria parasites field isolates that also further support its potential as a future antimalarial, given its mentioned efficacy against *P. falciparum*,^32^ particularly against liver stage forms,^74^ making it an ideal candidate for chemoprevention as well. Overall, these candidate therapeutics could be developed in clinical trials as efficacious tools for malaria further treatment. While this study provides important highlights of the susceptibility profiles of *P. falciparum* field isolates in Mali, a bigger sample size would give a better picture of current susceptibility profiles to the study drugs.

A key limitation of this study was the absence of molecular analyses to characterize *P. falciparum* resistance markers (*Pfmdr1, Pfcrt, Pfdhfr*, *dhps* and *Pfk13* mutations). This could have helped correlate parasite response to any mutation in the gene resistance markers. However, validated markers are not yet established for some compounds, such as tafenoquine, quinine and for next-generation compounds (cabamiquine, GNF179 and KDU691 in our study). Another important consideration is the potential confounding effect of folic acid administration, which may lead to the generation of field isolates with attenuated activity of antifolate drugs. Although this folate-related attenuation of SP efficacy is most clinically relevant in pregnant women receiving IPTp-SP, given the repeatedly administered doses of folic acid, it could have contributed to the elevated IC_50_ values for SP. This study was designed as a pilot investigation to inform a larger countrywide future study; therefore, the sample size was limited.

### Conclusions

Monitoring drug resistance spread is a key aspect to ensure that patients receive appropriate and effective treatment, as well as guiding public health policies towards data-driven, appropriate treatment. Thus, the surveillance is critical for real-time tracking of resistance patterns, which allows for the timely management and effective development of alternative interventions. This also guides the appropriate use of antimalarial drugs in settings with limited resources like Mali. The reduced susceptibility observed for withdrawn or unused drugs, such as tafenoquine and chloroquine, suggests that these compounds should be evaluated in larger and more representative sample sets in Mali, as monitoring of their susceptibility profiles remains epidemiologically valuable. The currently used drugs showed susceptible profiles with some significant increases in some strains’ IC_50_. Thus, the inter- and intra-variability in the profile of susceptibility for currently used drugs calls for enhancing continuous monitoring and engaging in developing new malaria treatments to overcome further drug resistance. The malaria candidate drugs displayed very low IC_50_, proving local isolate susceptibility prior to these drug candidates deployment as malaria therapies.
